# The Influence of Drying Time, Application Mode, and Agitation on the Dentin Bond Strength of a Novel Mesoporous Bioactive Glass-Containing Universal Dentin Adhesive

**DOI:** 10.3390/jfb16070247

**Published:** 2025-07-05

**Authors:** Jiyoung Kwon, Jungwon Kim, Dongseok Choi, Duck-Su Kim

**Affiliations:** 1 Department of Conservative Dentistry, Kyung Hee University Dental Hospital, Seoul 02447, Republic of Korea; jykt55@gmail.com; 2 Department of Conservative Dentistry, Armed Forces Capital Dental Hospital, Seongnam-si 13574, Republic of Korea; jw5875@gmail.com; 3 OHSU-PSU School of Public Health, Oregon Health & Science University, Portland, OR 97239, USA; choid@ohsu.edu; 4 Department of Conservative Dentistry, School of Dentistry, Kyung Hee University, Seoul 02453, Republic of Korea

**Keywords:** adhesive agitation, drying time, application mode, mesoporous bioactive glass, micro-tensile bond strength, universal adhesive

## Abstract

This study evaluated the influence of drying time, application mode, and agitation on the micro-tensile bond strength (μTBS) of a novel mesoporous bioactive glass-containing universal adhesive (Hi-Bond Universal). Twelve experimental groups were established according to drying time (blot-dry, 10 s dry, or 20 s dry), application mode (total-etch or self-etch), and agitation (with or without). The μTBS test and failure mode analysis were performed for each experimental group (*n* = 20), and an adhesive interface was observed using field-emission scanning electron microscopy. The μTBS of all experimental groups was analyzed using a three-way ANOVA and Tukey’s honestly significant difference (HSD) post hoc test (α = 0.05). The total-etch mode yielded higher μTBS than the self-etch mode in the blot-dry and 10 s dry groups (*p* < 0.05). Agitation also significantly increased the μTBS in the blot-dry and 10 s dry groups for both application modes (*p* < 0.05). However, application mode and agitation had no effect on the μTBS in the 20 s dry group (*p* > 0.05). FE-SEM revealed longer and more uniform resin tags after agitation in the blot-dry and 10 s dry groups for both application modes. In conclusion, total-etch mode and agitation effectively increased the bond strength of mesoporous bioactive glass-containing universal adhesives.

## 1. Introduction

Contemporary dentin adhesives can be applied using total-etch or self-etch mode. Ideal moisture control is important for both modes and is especially critical for the total-etch approach, where excessive drying can cause the collapse of the collagen network and negatively affect dentin adhesion. Nevertheless, maintaining appropriate dentin moisture is essential in self-etch systems, as both over-drying and excess wetness can negatively affect bond strength by altering the smear layer permeability and monomer diffusion [1,2]. Ideal moisture levels promote the infiltration of adhesive monomer into the dentinal tubules, thereby enhancing bond strength [3,4]. It also preserves collagen matrix integrity, reduces post-operative hypersensitivity, and guarantees reliable dentin bonding [5,6,7,8,9]. However, the clinical challenges posed by the variety of cavity configurations and the regional differences in dentin permeability—particularly between deep and superficial dentin—make it difficult to achieve ideal dentin moisture levels. Both excessive moisture in deeper dentin and excessive drying of dentin surfaces can significantly reduce bond strength, sometimes by 30–50%, thereby compromising the effectiveness of dentin bonding [10,11]. In routine practice, clinicians typically achieve ideal moisture by blot-drying the dentin surface. However, this process is technique-sensitive and subjective. A study showed that excessive drying and excess moisture can significantly reduce bond strength, emphasizing the importance of careful moisture control during adhesive procedures [12].

Bonding to dentin with various moisture levels leads to an unpredictable reduction in bond strength, regardless of the adhesive strategy or system [13]. In the total-etch mode, the reduced bond strength is primarily attributed to the collapse of the demineralized collagen matrix caused by over-drying. Structural collapse impairs adhesive monomer diffusion and infiltration into exposed collagen fibrils, resulting in inadequate hybrid layer formation and compromised micromechanical interlocking [14]. In the self-etch mode, the reduction of bond strength is related to the surface moisture of dentin and the composition of dentin adhesives [5,15,16].

Various problems associated with the non-ideal conditions of dentin moisture can be overcome by the agitation of dentin adhesives. This facilitates adhesive infiltration into dentin, forming a more stable adhesive layer. Various studies have evaluated the effects of adhesive agitation. Both ultrasonic and manual agitation techniques facilitate the deeper infiltration of resin monomers into the collagen network, thereby strengthening the hybrid layer and enhancing long-term bond durability when using a total-etch system [17]. Chan et al. further demonstrated that passive application of self-etch adhesives tends to trap the smear layer, whereas active agitation disperses or dissolves this layer, resulting in a more homogeneous hybridized zone [18]. Similarly, Velasquez et al. reported that agitation significantly increases the dentin shear bond strength of self-etch adhesives [19]. By promoting solvent evaporation and improving monomer penetration, agitation optimizes the immediate and sustained performance of both total- and self-etch protocols [20,21].

Recently developed universal adhesives represent a significant advancement in dental adhesive technology, as they are designed for versatility and can be applied using total-etch, self-etch, or selective-etch modes, providing clinicians with flexibility according to their clinical needs. These systems simplify the bonding procedure and reduce technique sensitivity, making them suitable for a wide range of restorative scenarios. A key component of most universal adhesives is 10-methacryloyloxydecyl dihydrogen phosphate (10-MDP) monomer, which plays a crucial role in the bonding mechanism [22]. This component chemically interacts with hydroxyapatite in the tooth structure, forming stable MDP-Ca salts that enhance the durability and strength of the adhesive interface. This chemical interaction contributes to improved long-term bond stability and resistance to hydrolytic degradation [23].

Building on advancements in adhesive chemistry, recent efforts have focused on integrating bioactive materials such as bioactive glass (BAG) into dental adhesives. Since its introduction by Hench in 1969, BAG has been valued for its ability to form a silica-rich layer and induce hydroxycarbonate apatite formation in physiological environments, thereby supporting hard-tissue mineralization [24]. Sol–gel-synthesized BAG has demonstrated additional therapeutic benefits, including the reduction of dentin hypersensitivity, through its ability to occlude dentinal tubules and promote mineral deposition [25,26]. Improvements in sol–gel processing have yielded mesoporous bioactive glass (MBG) with a dramatically increased surface area and pore volume, enabling a more rapid and sustained release of calcium and other therapeutic ions than conventional BAG. This mesostructure not only augments mineralization and mechanical properties but also accelerates hydroxyapatite layer formation at the resin–dentin interface, enhancing restoration durability. A novel MBG-incorporated universal adhesive (Hi-Bond Universal; MEDICLUS, Cheongju, Korea) has recently been introduced to capitalize on these properties; however, the effect of agitation on its bonding performance under various conditions remains unclear.

Therefore, this study aimed to evaluate the effects of drying time, application mode, and agitation on the bond strength of Hi-Bond Universal. The following null hypotheses were evaluated: (1) drying time does not substantially increase dentin bond strength, (2) application mode does not substantially increase dentin bond strength, and (3) agitation of Hi-Bond Universal does not substantially increase dentin bond strength.

## 2. Materials and Methods

### 2.1. Study Design

**Figure 1:**
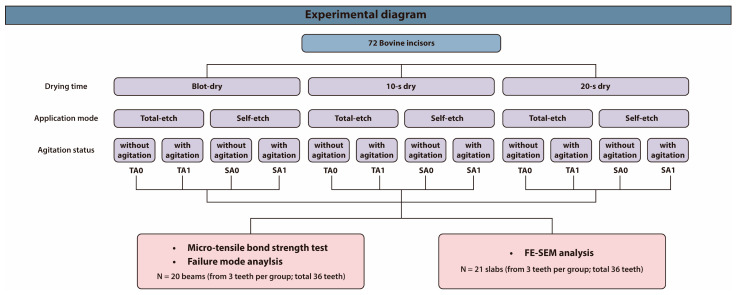
Experimental diagram of the study. Seventy-two bovine incisors were allocated to 12 experimental groups based on three drying processes, two application modes, and two agitation conditions. Abbreviations: A0, without agitation; A1, with agitation; T, total-etch; S, self-etch; FE-SEM, field-emission scanning electron microscopy.

This in vitro study employed a 3 × 2 × 2 factorial design to evaluate the effects of three variables—drying time, application mode, and agitation—on the micro-tensile bond strength (μTBS) of Hi-Bond Universal. The drying time was categorized into three groups: blot-dry, 10 s dry, and 20 s dry. The application mode was set as either total-etch (T) or self-etch (S), and agitation was performed either without (A0) or with (A1) active agitation. This design resulted in 12 experimental groups. The detailed experimental protocols are provided in Table 1, and an overall experimental diagram is illustrated in Figure 1.

### 2.2. Micro-Tensile Bond Strength (μTBS) Assessment

Thirty-six bovine incisors, freshly extracted and purchased from a local market, were selected as substrate specimens, prepared to standardized dimensions, and randomly allocated to 12 experimental groups. The use of bovine teeth was predicated on their well-documented equivalence to human dentin in terms of physical and mechanical properties, particularly in bond strength testing paradigms. This approach aligns with the conclusions of a systematic review conducted by Soares et al. [27], who found no statistically significant differences in adhesive performance between bovine and human dentin substrates when standardized protocols were followed. The sample size was determined based on previous studies using G*Power software (version 3.1.9.7) [28,29]. An effective sample size of 20 subjects per group was calculated to achieve a statistical power > 0.95 (β = 0.05) with a significance level (α) of 0.05.

The root portion was cut, and the fresh dentin surface was exposed using diamond burs. The dentin surfaces were polished in ascending order with 180-, 320-, and 600-grit silicon carbide paper, with each grit applied for 30 s to standardize the smear layer. The prepared teeth were randomly assigned to groups based on drying time (blot-dry, 10 s dry, 20 s dry), application mode (total-etch [T] or self-etch [S]), and agitation (without [A0] or with [A1]).

The “10 s dry” method was chosen to reflect common clinical conditions, as it effectively evaporates the solvent without causing significant collagen collapse, thus preserving bond strength [2,12]. The “20 s dry” method simulates over-drying, which has been shown to lead to irreversible collagen collapse and significantly reduced bond strength [30,31].

Three different drying times were defined as follows: for the blot-drying process, excess moisture was air-dried for 2 s using a three-way syringe at 1 bar of pressure, positioned at a 45° angle and 1.5 cm from the dentin surface to simulate clinically relevant drying to produce a moist surface; for the 10 s drying process, air-drying was performed for 10 s; and for the 20 s drying process, air-drying was conducted for 20 s to simulate an over-drying condition of the dentin surface [2].

A droplet of Hi-Bond Universal was applied to each specimen using a micro-brush. For the total-etch, dentin surfaces were treated with 35% phosphoric acid (Select HV Etch; BISCO, Schaumburg, IL, USA) for 15 s, followed by thorough rinsing with water for 30 s using a three-way syringe. Subsequently, the adhesive was applied to the prepared dentin surface with or without agitation for 10 s. In the self-etch group, the adhesive was applied directly to the dentin surfaces with or without agitation for 10 s. Following adhesive application, all specimens underwent photopolymerization for 20 s using an LED (light-emitting diodes) curing light (Bluephase 20i; Ivoclar; Schaan, Liechtenstein) with an intensity of 1200 mW/cm^2^. A 4 mm thick composite resin (Any-Com; MEDICLUS, Cheongju, Korea) was incrementally applied and subjected to a 20 s photopolymerization cycle using the same LED curing unit. After polymerization, all specimens were immersed in distilled water at 37 °C for 24 h. Table 2 lists the compositions of the materials used in this study.

After fabrication, all specimens were stored in distilled water at 37 °C for 24 h before μTBS test. Each specimen was then sectioned into 1 mm thick slabs using a high-speed diamond saw (IsoMet 5000; Buehler Ltd., Lake Bluff, IL, USA). These slabs were further trimmed to yield 1 × 1 mm^2^ composite–dentin beams according to the non-trimming μTBS protocol [32]. For each group, 20 beams were randomly selected to ensure representative sampling. Each beam was carefully bonded to a μTBS jig using cyanoacrylate adhesive (Loctite 454^®;^ Henkel, Düsseldorf, Germany) and then mounted on a universal testing machine (AGS-X; Shimadzu, Tokyo, Japan). A tensile load was applied at a crosshead speed of 1 mm/min until failure, and the peak stress at fracture was recorded.

### 2.3. Failure Mode Analysis

Following the μTBS measurement, the fractured surface of each specimen was analyzed using an optical stereomicroscope (SZN745; Sunny, Shanghai, China) at 40× magnification to determine the failure modes. The failure patterns were classified into three categories: (1) adhesive failure (occurring within the adhesive layer), (2) mixed failure (combining adhesive and cohesive failures across the bonded interface), and (3) cohesive failure (occurring within the underlying dentin or composite layers).

### 2.4. Field-Emission Scanning Electron Microscopy (FE-SEM) Analysis of the Bonded Interfaces

Specimens for FE-SEM analysis were prepared as described for the μTBS tests, using three bovine incisors per experimental group. To examine the adhesive interface between the composite resin and dentin, each tooth was sectioned vertically into seven slabs using a high-speed diamond saw (IsoMet 5000). Specimens were processed according to Perdigao et al. and then analyzed using FE-SEM (Apreo S; Bruker, Billerica, MA, USA) [33].

### 2.5. Statistical Analysis

To determine the association between drying time, application mode, and agitation, the results of the μTBS test were assessed using a three-way ANOVA. Post hoc analysis was conducted using Tukey’s honestly significant difference (HSD), with *α* set at 0.05. All computations were performed using the R software (version 4.4.0) and GraphPad Prism 10.4.0 (GraphPad Software Inc., San Diego, CA, USA).

## 3. Results

### 3.1. μTBS Assessment

**Figure 2:**
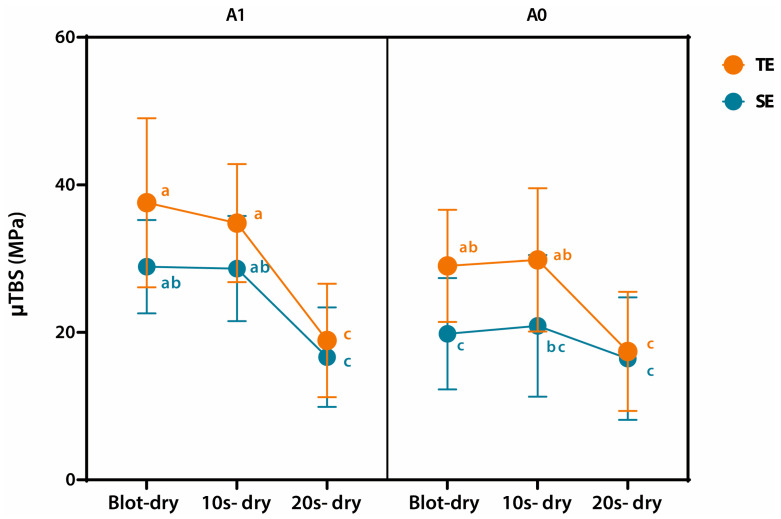
Mean micro-tensile bond strength (µTBS) values expressed in megapascals (MPa) for each group according to dentin moisture conditions. Specimens were grouped according to drying time (blot-dry, 10 s dry, and 20 s dry), application mode (total-etch [T] or self-etch [S]), and agitation (A0 = without agitation; A1 = with agitation). Error bars indicate standard deviation. Same lowercase letters indicate statistically significant differences among application modes and agitation within the same degree of moisture (two-way analysis of variance [ANOVA], Tukey’s honestly significant difference [HSD], α = 0.05).

The μTBS values are shown in Figure 2. A three-way ANOVA was performed to conduct the overall analysis and to evaluate the effects of drying time, application mode, and agitation on the bond strength of the adhesive. Correlation analysis revealed significant associations between drying time and agitation, as well as between drying time and etching mode (*p* = 0.0052 and 0.0171, respectively; Table 3). Notably, when the dentin was dried for 20 s, the bond strength was markedly reduced, regardless of the agitation procedure or application mode applied. The three-way ANOVA confirmed that the main effect of drying time was dominant, and the presence of significant correlations between moisture and the other two factors further supports the interpretation that excessive drying is a key determinant of reduced bond strength, overriding the effects of agitation and etching mode under dry conditions. These results suggest that maintaining appropriate dentin moisture is essential, as drying the dentin surface consistently leads to weak bonding, regardless of additional procedural variations. Three-way ANOVA showed no significant differences and no significant interaction between factors in the 20 s dry group (Figure 2).

### 3.2. Failure Mode Analysis of the Fractured Surfaces

**Figure 3:**
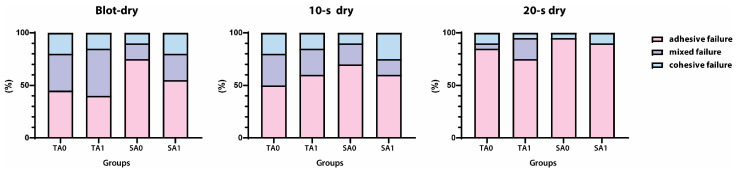
Percentage of each type of failure mode in each group according to dentin moisture conditions. Failure patterns are classified as adhesive (failure at the adhesive–dentin interface), mixed (combination of adhesive and cohesive failures), or cohesive (failure within the dentin or composite). Groups are arranged by drying time (blot-dry, 10 s dry, and 20 s dry), application mode (total-etch [T] or self-etch [S]), and agitation (without [A0] or with [A1]).

Figure 3 shows the distributions of the failure patterns in each experimental group. Most specimens predominantly exhibited adhesive and mixed failures, with a low incidence of cohesive failure. In the blot-dry groups, mixed failure increased with agitation, regardless of the application mode. The 10 s dry group showed no relevant changes in failure mode. However, the 20 s dry group exhibited a notable increase in adhesive failure.

### 3.3. FE-SEM Analysis of the Bonded Interfaces

**Figure 4:**
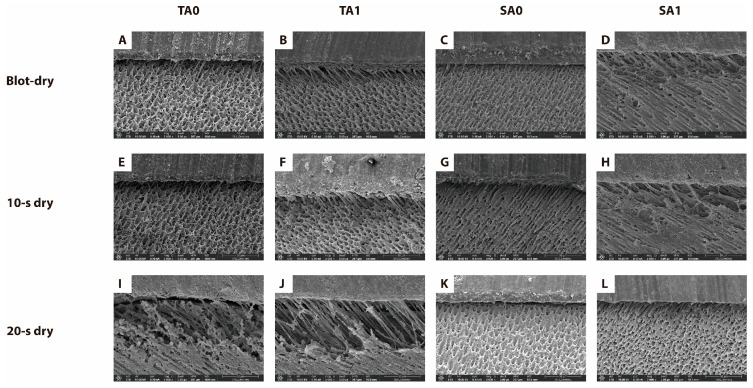
FE-SEM micrographs of the resin–dentin interfaces in all groups (magnification × 2000). The scale bar represents 50 μm. (**A**–**D**) Blot-dry: (**A**) TA0, (**B**) TA1, (**C**) SA0, and (**D**) SA1; (**E**–**H**) 10 s dry: (**E**) TA0, (**F**) TA1, (**G**) SA0, and (**H**) SA1; (**I**–**L**) 20 s dry: (**I**) TA0, (**J**) TA1, (**K**) SA0, (**L**) and SA1. Agitation produced longer and more uniform resin tags (**B**,**D**,**F**,**H**,**J**,**L**), whereas the non-agitated 20 s dry group (**I**,**K**) exhibited partially detached or collapsed hybrid layers. FE-SEM, field-emission scanning electron microscopy; T, total-etch; S, self-etch; A0, without agitation; A1, with agitation.

The results of the bonded interfaces are shown in Figure 4. The blot-dry and 10 s dry groups showed intact adhesive layers with underlying dentin in both the total-etch and self-etch modes (Figure 4A–I). Longer resin tags were observed in the hybrid layer after agitation, regardless of the application mode (Figure 4B,D,F,H). However, partially detached hybrid layers were observed in the TA0 and SA0 20 s dry groups (Figure 4I,K). After agitation, the hybrid layers remained intact, and longer resin tags were formed (Figure 4J,L).

## 4. Discussion

This study evaluated the dentin bond strength of a novel MBG-containing universal adhesive under different drying times, application modes, and agitation conditions. Based on the results of this study, the first null hypothesis was rejected. Additionally, both application mode and agitation increased bond strength in the blot-dry and 10 s dry groups, but not in the 20 s dry group; thus, the second and third null hypotheses were partially rejected.

In the present study, we used Hi-Bond Universal, a novel commercial dentin adhesive that contains MBG for tooth remineralization, as stated by the manufacturer. MBG, a BAG with a particle size of 2–50 nm, exhibits superior bioactivity compared to conventional melt-quenched BAG. While recent studies have demonstrated the positive effects of BAG-containing dentin adhesives, their findings are somewhat limited because they primarily involved experimental adhesives rather than commercial products [34,35,36]. In contrast, this study utilized the commercially available Hi-Bond Universal adhesive, providing more clinically relevant data. Although we did not directly compare the bond strength among different universal dentin adhesives, a previous systematic review reported that the μTBS of various universal adhesives ranges from 20 to 50 MPa [37]. Notably, since the test methods for μTBS were not identical across studies, the μTBS values for Hi-Bond Universal under blot-dry and 10 s dry conditions in our study were still within the reported range.

This study evaluated three drying processes: blot-dry, 10 s dry, and 20 s dry. Blot-dry is an ideal method for dentin bonding. Although they can be evenly simulated in vitro, achieving blot-dry conditions in clinical settings remains challenging. Therefore, a 10 s dry was used considering various factors such as cavity configuration, dentin location (superficial, middle, and deep), and the distance between the cavity and air stream, which may be encountered in clinical situations. Therefore, in a clinical setting, the practical drying time is between the blot-dry and 10 s dry times, whereas 20 s dry is a severe condition for dentin bonding.

Air-drying affects dentin bonding by altering the surface microstructure. After caries removal or cavity preparation, a smear layer is formed. This thin, loose, and amorphous layer of debris—comprising organic and inorganic materials, including hydroxyapatite particles, collagen fragments, and occasionally bacteria or their byproducts—forms on dentin or enamel surfaces. This layer can occlude dentinal tubules and interfere with adhesion unless properly managed during bonding procedures. In the total-etch mode, the smear layer is usually removed by acid etching, which exposes a demineralized collagen fiber network. Over-dry can cause collagen collapse, which interferes with the infiltration of adhesive monomers. In the self-etching approach, the smear is either partially dissolved or modified. If the self-etched dentin surface is excessively dried, the loose smear layer becomes densely packed. Therefore, modifying self-etching adhesives with acidic functional monomers is challenging.

The results of the present study demonstrate similar μTBS values between the blot-dry and 10 s dry groups. Therefore, drying times of 2–10 s did not adversely affect the dentin bond strength of the Hi-Bond Universal adhesive. At these drying times, the total-etch groups exhibited higher μTBS values than the self-etch groups. This outcome is consistent with the findings of previous studies showing that the total-etch mode effectively removes the smear layer from both enamel and dentin surfaces [38]. The increased micromechanical interaction after separate etching allows for better adhesive penetration and a more robust hybrid layer, producing stronger and more durable bonds [39]. Conversely, self-etch adhesives tend to modify, rather than completely remove, the smear layer, which can limit adhesive infiltration and compromise bond strength, particularly in thicker and more resilient smear layers. Therefore, our finding of a higher bond strength in the blot-dry and 10 s dry groups using the total-etch approach can be attributed to optimized surface moisture and adhesive infiltration.

The characteristics of the smear layer have diverse effects on bond strength in the self-etch approach. According to the manufacturer, the pH of Hi-Bond Universal is approximately 3.2. It is classified as a mild or ultra-mild dentin adhesive; therefore, its bonding performance is particularly influenced by the presence and characteristics of the smear layer when using the self-etch approach. Additionally, the smear layer formed by silicon carbide paper may be thicker than that formed by diamond burs [40]. A thicker smear layer may also contribute to the lower bond strength in the self-etch mode.

Agitation increased μTBS in both the blot-dry and 10 s dry groups, regardless of the application mode. For the total-etch, it mechanically compresses collapsed collagen, which then re-expands upon pressure release and draws resin deep into the dentin matrix, reinforcing the hybrid layer [41]. It also enhances solvent diffusion and improves the penetration of adhesives. Although it does not change hydrophilicity, agitation minimizes nanopore formation within the polymer network, thereby increasing the cross-link density. The resulting denser and more cohesive adhesive matrix exhibits superior mechanical properties, yielding a stronger, more durable bond and long-term stability [20,42]. In self-etch mode, agitation improved the μTBS by modifying the smear layer, which facilitates acidic monomer access to hydroxyapatite and accelerates solvent evaporation. Senawongse et al. demonstrated that agitation enhanced bond strength even with a thicker smear layer [43], and Amaral et al. reported similar results across three single-step self-etch adhesives [44]. However, in the 20 s dry group, μTBS decreased markedly, and agitation had no effect. Kumagai et al. attributed this to irreversible collagen collapse because of over-drying in total-etch systems and excessively dense smear layers in self-etch protocols, both of which, despite agitation, hinder hybrid layer formation [30]. Furthermore, regardless of agitation, the dentin surface was not favorable for proper bonding.

The fracture mode analysis also supported the results of the μTBS test. Adhesive fractures were more predominant and numerous in the 20 s dry group than in the blot-dry and 10 s dry groups. At the bonded interface, the adhesive layer, including the hybrid layer, is typically weak because it comprises fully or partially demineralized dentin and an unfilled adhesive. The 20 s dry condition rendered the dentin surface unfavorable for bonding, an effect that could not be overcome by agitation. Consequently, the specimens in this group were the most susceptible to fracture during the μTBS test.

FE-SEM analysis provided further evidence. Regardless of the application mode, adhesive agitation increased adhesive monomer penetration, forming longer resin tags. Agitation increased μTBS in the blot-dry and 10 s dry group, but not in the 20 s dry group. Severe collagen collapse was observed in all etching groups (Figure 4I,J). Despite the longer resin tags after agitation, proper hybridization was not achieved. Thus, agitation cannot entirely compensate for unfavorable conditions like a 20 s dry time. In self-etch mode, although agitation resulted in a more intact hybrid layer, μTBS did not increase. Therefore, other chemical reactions that could not be found by FE-SEM may have occurred.

This study successfully demonstrated the effects of agitation and etching on the bond strength of a novel MBG-containing universal dentin adhesive under various drying times. These findings provide valuable insights into the influence of these factors on adhesive interactions and bond strength. However, this study had some limitations that should be acknowledged. First, all experiments were conducted in vitro, using bovine incisors as substitutes for human teeth. Although previous research [27] has shown that bovine and human dentin have comparable physical properties and are commonly used interchangeably in bond strength tests, subtle structural and compositional differences may exist, potentially limiting the generalizability of these findings. Second, the specimens were stored in distilled water for only 24 h prior to testing, and no artificial aging procedures, such as thermocycling or mechanical loading, were performed. The absence of long-term storage or aging protocols means that the results reflect only the immediate bond strength and do not provide information on the durability of the adhesive interface over time. Therefore, further studies using human tooth specimens and incorporating long-term aging protocols are necessary to confirm the clinical applicability and long-term performance of these findings.

## 5. Conclusions

The findings of this study provide valuable information on achieving reliable dentin bond strength using a novel MBG-containing universal adhesive. An appropriate drying time of 2–10 s, using the total-etch technique rather than the self-etch technique, and agitation of the adhesive constitute promising options for stable adhesion. These results highlight the need for precise moisture control during restorative procedures to ensure reliable and durable dental restorations.

## Figures and Tables

**Table 1:** Experimental protocol of this study.

Groups	Subgroup	Procedure
Drying time	Blot-dry	The dentin surface was air-dried for 2 s with a three-way syringe at a pressure of 1 bar, with the air nozzle positioned at a 45° angle and 1.5 cm away from the dentin surface.
	10 s dry	The dentin surface was air-dried for 10 s with a three-way syringe at a pressure of 1 bar, with the air nozzle positioned at a 45° angle and 1.5 cm away from the dentin surface.
	20 s dry	The dentin surface was air-dried for 20 s with a three-way syringe at a pressure of 1 bar, with the air nozzle positioned at a 45° angle and 1.5 cm away from the dentin surface.
Application mode	Total-etch	The dentin surfaces were treated with 35% phosphoric acid for 15 s, followed by thorough water rinsing for 30 s with a three-way syringe.
	Self-etch	The adhesive was applied directly to the dentin surfaces.
Agitation	With agitation	The adhesive was agitated actively for 10 s with a micro-brush under manual pressure.
	Without agitation	The adhesive was applied passively for 3 s with a micro-brush and left undisturbed for 5 s.

**Table 2:** Compositions of the materials used in the study.

Product	Composition
Hi-Bond Universal (MEDICLUS, Cheongju, Republic of Korea)	Mesoporous bioactive glass, 10-MDP, Bis-GMA, HEMA, water, ethanol, silane coupling agent, photo-initiator, accelerators, etc.
Any-Com (MEDICLUS, Cheongju, Republic of Korea)	Bis-GMA, UDMA, TEGDMA, barium glass, silicon dioxide, photo-initiators, accelerators, other additives.

Abbreviations: 10-MDP, 10-methacryloyloxydecyl dihydrogen phosphate; Bis-GMA, Bisphenol A-glycidyl methacrylate; HEMA, 2-Hydroxyethyl methacrylate; UDMA, urethane dimethacrylate; TEGDMA, triethylene glycol dimethacrylate.

**Table 3:** Results of three-way ANOVA test showing F values and levels of significance (*p* value).

ANOVA Table	F Value	*p* Value
Drying time	53.82	<0.0001
Agitation	29.70	<0.0001
Application mode (Mode)	31.63	<0.0001
Drying time × Agitation	5.779	0.0052
Drying time × Mode	4.375	0.0171
Agitation × Mode	0.08004	0.7783
Drying time × Agitation × Mode	0.2454	0.7832

Abbreviations: ANOVA, analysis of variance.

## Data Availability

The data presented in this study are available upon request from the corresponding author.

## Abbreviations

The following abbreviations are used in this manuscript:

A0	Without agitation
A1	With agitation
ANOVA	Analysis of variance
BAG	Bioactive glass
FE-SEM	Field-emission scanning electron microscopy
HCA	Hydroxycarbonate apatite
HSD	Honestly significant difference
MBG	Mesoporous bioactive glass
MPa	Megapascal
μTBS	Micro-tensile bond strength
S	Self-etch
SEM	Scanning electron microscopy
T	Total-etch
